# Medial to lateral diagonal injury of the elbow without elbow dislocation in children

**DOI:** 10.3389/fped.2026.1762246

**Published:** 2026-03-05

**Authors:** Limin Hou, Jie Li, Wei Zhang, He Hu, Peng Yue, Peng Wang, Fei Jiang

**Affiliations:** 1 Department of Pediatric Orthopaedic, Dalian Women and Children's Medical Group, Dalian City, Liaoning Province, China; 2 Department of Pediatric Orthopaedic, Shanxi Children's Hospital, Taiyuan, China; 3 Department of Neonatology, Dalian Women and Children's Medical Group, Dalian City, Liaoning Province, China

**Keywords:** children, diagonal fracture, medial epicondyle fracture, the carrying angle, without elbow dislocation

## Abstract

**Purpose:**

The incidence of medial to lateral diagonal elbow injury (MELAINE) in children without dislocation is relatively low. This article explores the clinical characteristics and treatment outcomes of this fracture type.

**Methods:**

A retrospective analysis of elbow fracture was performed in Dalian Women and Children's Medical Center (Group) and Shanxi Provincial Children's Hospital, between January 2019 and January 2025. The collected data encompassed patient age, gender, side, diagnose. Additional parameters included the injury mechanism, elbow joint range of motion, and radiological findings. We also recorded the carrying angle (CA), Elbow Performance Scale (EPS) score, treatment method, healing time, and complications.

**Results:**

The study included 21 patients (2.21%, 21/949), mean age 9.4 ± 3.08 years (range 8–14 years); 14 males and 7 females, 13 left and 8 right, from injury to surgery was 3.1 days (range 1–11 days), and the mean follow-up duration was 11.46 ± 1.73 months (range 6–37 months). 8 extension-type and 13 flexion-type. All patients underwent open reduction and internal fixation, performed via a medial approach in 19 cases, a lateral approach in 10 cases, and a combined medial and lateral approach in 8 cases. At final follow-up, the mean elbow flexion–extension and forearm pronation–supination arcs on the fractured side were 139.2° ± 9.4°, 4.5° ± 3.4°, 75.8° ± 8.1°, and 79.6° ± 8.2°, respectively, showing no significant difference from the healthy side (*p* > 0.05). The carrying angle on the injured side measured 14.3° ± 2.8°, respectively, compared to 15.1° ± 1.7° on the healthy side (*p* = 1.78). According to the EPS rating, most patients achieved an “excellent” (*n* = 18, 85.7%) or “good” (*n* = 3, 14.3%) outcome.

**Conclusion:**

In older children and adolescents, medial-to-lateral diagonal elbow fractures without dislocation may be missed. The fractures frequently involve significant displacement of the medial epicondyle, yet surgical intervention can often achieve favorable clinical outcomes.

## Introduction

Pediatric elbow fractures account for approximately 5% to 10% of all childhood fractures (1–4). Common isolated patterns include fractures of the medial humeral epicondyle (MEP, ∼10%), fractures of the lateral humeral condyle (LC, 17%–34%), and fractures of the proximal radius, including radial neck injuries (RN, 5%–10%). Fractures of the capitellum are relatively infrequent, with an incidence of roughly 1%.

Combined elbow fractures are relatively uncommon in the pediatric population. Lacey et al. report a series of elbow dislocations in children (5), the fractures of the medial epicondyle were the most frequent (65%, 17/26), the lateral condyle (31%, 8/26) and the proximal radius (27%, 7/26). Combined fractures without elbow dislocation are even scarcer. Previous studies have described two patterns of pediatric “diagonal” fractures without elbow dislocation: the lateral-to-medial pattern (LAMEINE), involving the lateral condyle with an olecranon fracture (6), and the medial-to-lateral pattern (MELAINE) (7), comprising a medial epicondyle fracture with a radial neck fracture. We identified a series of the medial epicondyle Combined with either the lateral condyle or capitellum fracture. This article evaluates the radiological characteristics and clinical outcomes of this fracture pattern.

Combined elbow fractures are uncommon in pediatric population. In a series cases reported by Lacey et al., fractures of the medial epicondyle were the most frequent (65%, 17/26), followed by fractures of the lateral condyle (31%, 8/26) and the proximal radius (27%, 7/26) in children with elbow dislocations (5). Combined fractures without elbow dislocation are even scarcer in older children and adolescents. Previous studies have described two patterns of pediatric “diagonal” fractures without dislocation: the lateral-to-medial pattern (LAMEINE), which involves the lateral condyle with an olecranon fracture (6), and the medial-to-lateral pattern (MELAINE), comprising a medial epicondyle fracture with a radial neck fracture (7). We identified a series of fractures involving the medial epicondyle combined with lateral condyle or capitellar fracture. This article evaluates the imaging characteristics and clinical outcomes of this fracture pattern.

## Materials and methods

This research has been approved by the Ethics Committee of Dalian Women and Children's Medical Group.. The retrospective analysis was conducted on elbow fracture cases from two centers: Dalian Women and Children's Medical Center (Group) and Shanxi Provincial Children's Hospital, from January 2019 to January 2025. The data included age, gender, side (left/right), fracture location, injury mechanism, range of motion of the elbow joint, radiological data, the carrying angle (CA), the Elbow Performance Scale (EPS) score, treatment method, healing time, and complications.

Inclusion criteria: (1) age <16 years; (2) a unilateral elbow joint fracture; (3) without concomitant elbow dislocation; (4) complete clinical and imaging data; (5) a follow-up period exceeding six months. Exclusion criteria: (1) pathological or open fractures.

The MEP fractures were classified using the Papavasiliou system (8). The RN fractures were classified according to the system described by Judet et al. (9). The lateral condyle of the humerus were classified according to Song classification (10).

All images were reviewed by fellowship-trained orthopedic surgeon Peng Yue and surgeon Peng Wang with more than 5 years of experience in treating pediatric fractures. All the distances and angles were measured by a fellowship-trained orthopedic surgeon Limin Hou, and Jie Li using the same workstation with more than 10 years of experience in treating pediatric fractures. Discrepancies were resolved by consensus and adjudication by a third reviewer Fei Jiang, with 20 years of experience in treating pediatric fractures.

### Treatment

All surgeries were performed under general anesthesia in the conventional supine position by doctors with over ten years of experience in pediatric orthopedic trauma treatment. For song classification type 4/5 lateral condylar fractures of the humerus, closed or open reduction was followed by internal fixation with 2 or 3 Kirschner wires. For radial neck fractures, closed reduction and internal fixation with intramedullary pins or no fixation was adopted. For type 2/3/4 fractures of the medial epicondyle of the humerus, open reduction and internal fixation with Kirschner wires or absorbable screws was performed. The stability or dislocation of the elbow joint was checked both before and after the operation. Other conditions were treated conservatively with long-arm plaster external fixation for 4 to 6 weeks.

### Statistical analysis

Datas were analyzed using SPSS 26.0: Normally distributed variables: mean ± SD (ANOVA/t-test for intergroup comparisons). Non-normally distributed variables: median (IQR) (rank-sum test). Categorical data: percentages (chi-square/Fisher's exact test). Bonferroni correction for pairwise comparisons; *p* < 0.05 denoted significance (two-sided).

## Results

**Figure 1:**
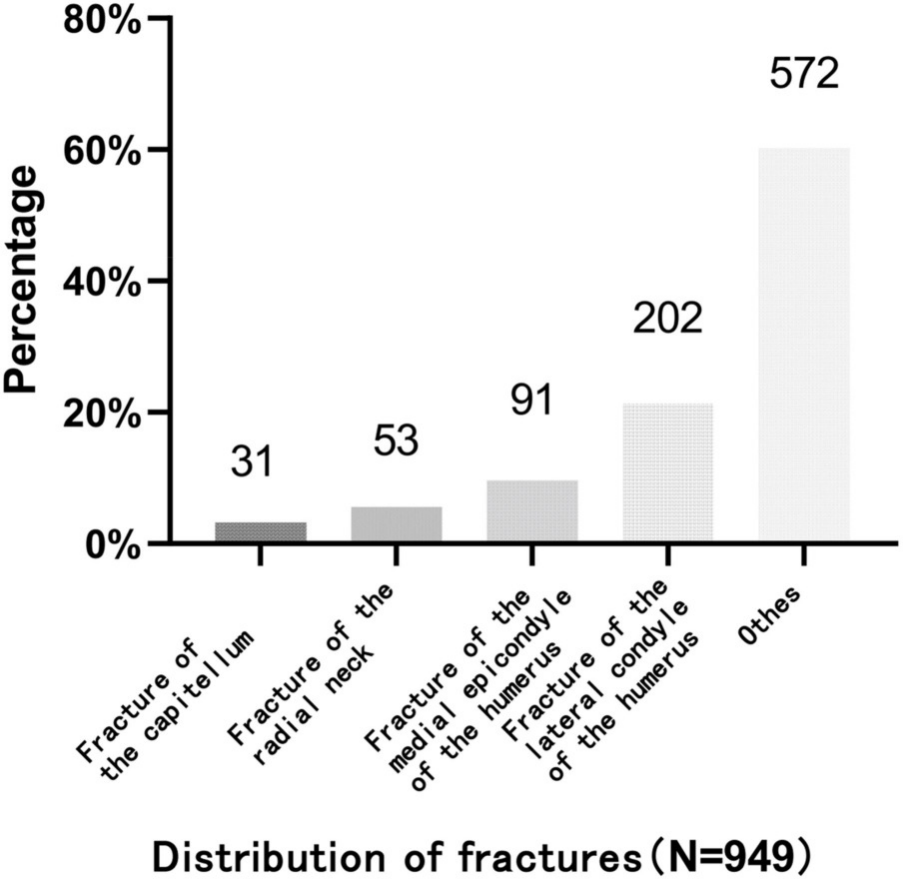
Distribution of fractures.

A total of 21 patients (2.21%, 21/949) were included, with a mean age of 9.4 ± 3.08 years (range 8–14 years); male 14 and female7, left 13 and right 8. The mean interval from injury to surgery was 3.1 days (range 1–11 days), and the mean follow-up duration was 11.46 ± 1.73 months (range 6–37 months). Injury mechanisms included traffic accidents (*n* = 2), falls from height (*n* = 3), fall down (*n* = 9), sports injuries (*n* = 6), and collision (*n* = 1) (Figure 1).

**Figure 2:**
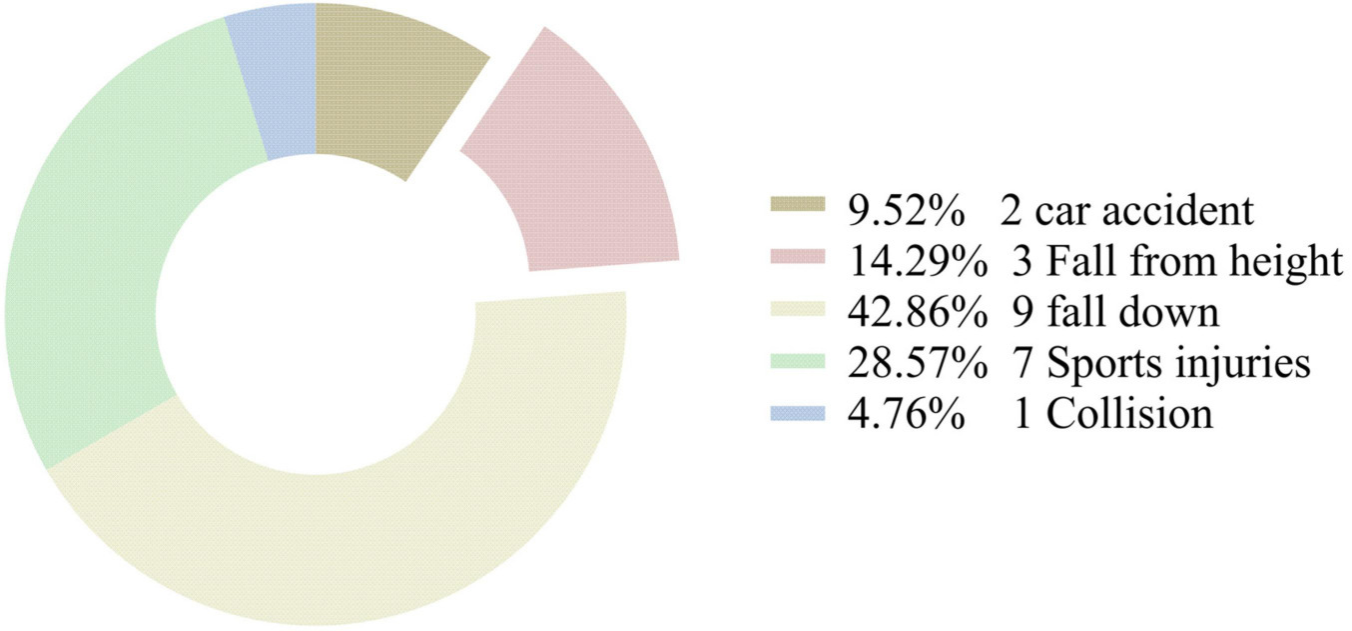
Distribution of Injury mechanisms.

Among the 949 cases, MEP accounted for 9.59% (91/949), RN 5.58% (53/949), LC 21.30% (202/949), and capitellar fractures 3.20% (31/949). The cohort comprised 21 patients with MELAINE, classified as extension-type (*n* = 8) and flexion-type (*n* = 13); among these, MEP were classified as type II (*n* = 5) and type III (*n* = 16). RN were classified as type II (*n* = 2), type III (*n* = 3), and type IV (*n* = 3); LC (*n* = 10) were classified as type III (*n* = 4), type IV (*n* = 2), and type V (*n* = 4). All patients underwent open reduction and internal fixation via a medial operative route in 19 cases, a lateral operative route in 10 cases, and a combined medial and lateral operative route in 8 cases (Figure 2).

**Figure 3:**
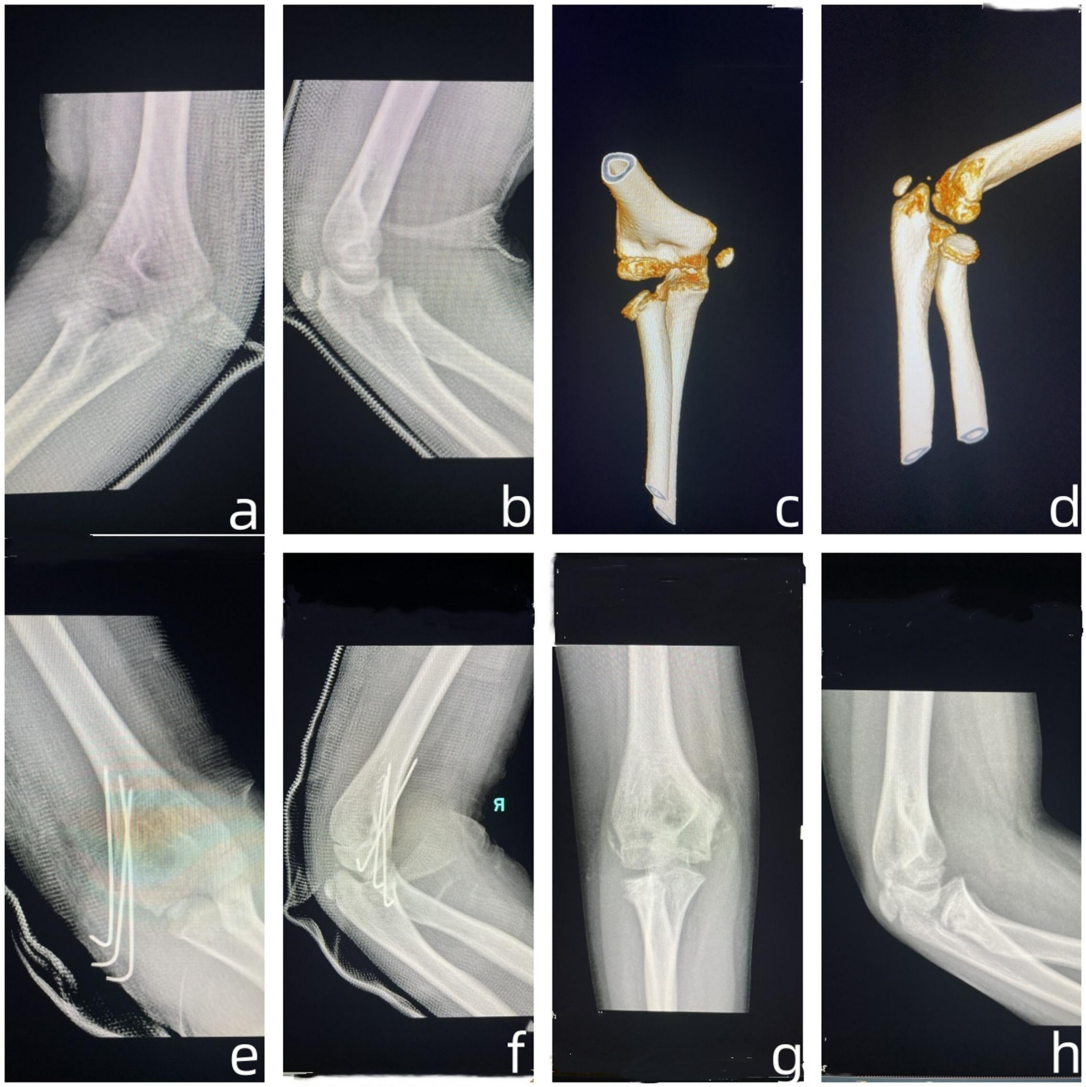
**(a)** One 10 years boy, fall from height, 1 day after injury the Anteroposterior radiographs; **(b)** 1 day after injury the Lateral radiographs; **(c)** 2 day after injury the Anteroposterior Three dimensional CT; **(d)** 2 day after injury the Lateral Three dimensional CT; **(e)** 1 day post operation the Anteroposterior radiographs; **(f)** 1 day post operation the Lateral radiographs; **(g)** 3 months post operation the Anteroposterior radiographs; **(h)** 3 months post operation the Lateral radiographs.

The MELAINE-extension type comprised eight cases (male 5, female3), left side 6 and right side 2. Injury mechanism included one car accident, two falls from height, three simple falls, one sports injury, and one collision. All patients underwent surgical open reduction. This group included eight MEP (type II 2, type III 6), fixed with absorbable screw1 and Kirschner wires 7. It also included 8 RN, type II (*n* = 2), type III (*n* = 3), and type IV (*n* = 3), all treated with close reduction; fixed with intramedullary nailing 5 and without fixed 3 (Figure 3).

**Figure 4:**
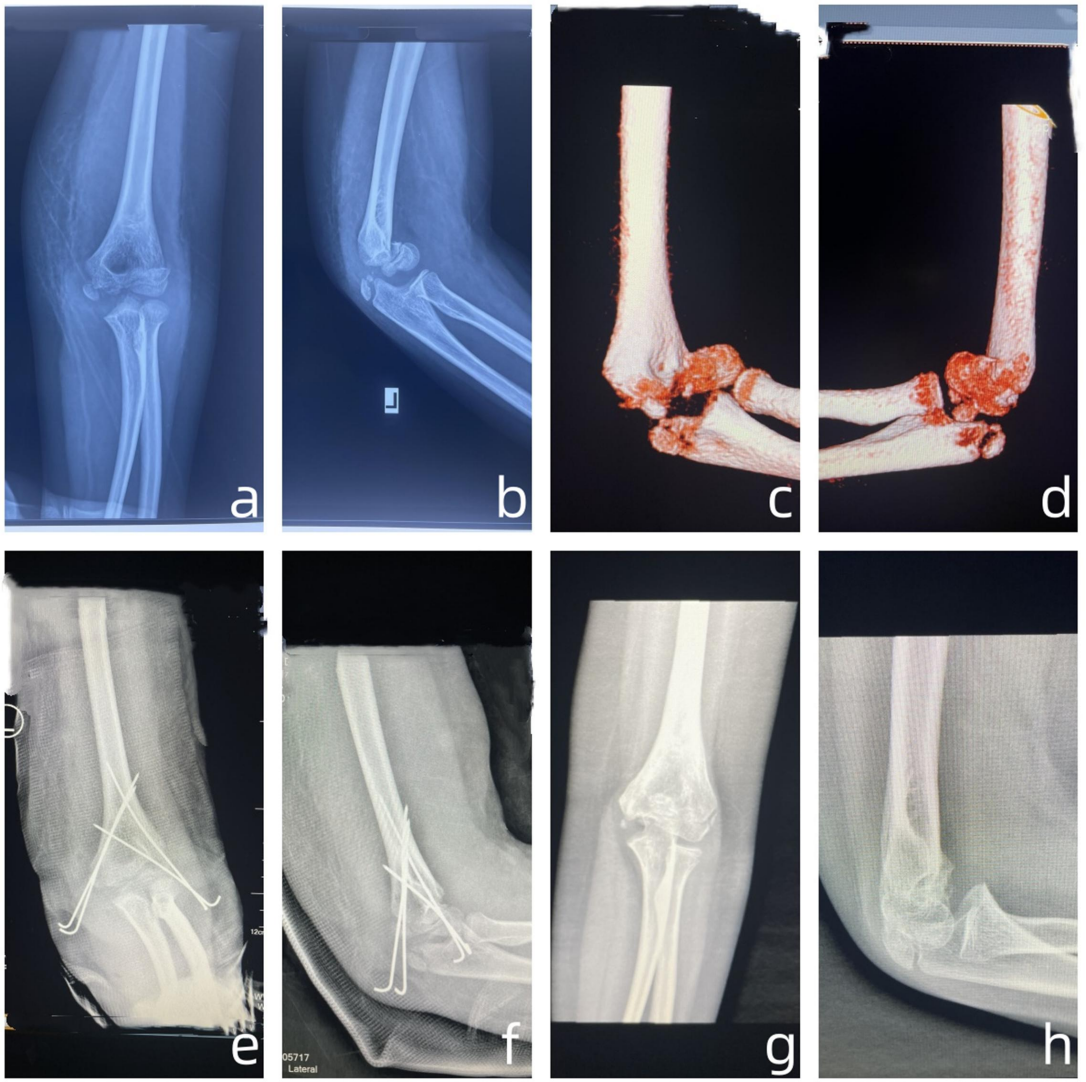
**(a)** One 10 years boy, sports injuries, 1 day after injury the Anteroposterior radiographs; **(b)** 1 day after injury the Lateral radiographs; **(c)** 1 day after injury the Anteroposterior Three dimensional CT; **(d)** 1 day after injury the Lateral Three dimensional CT; **(e)** 1 day post operation the Anteroposterior radiographs; **(f)** 1 day post operation the Lateral radiographs; **(g)** two months post operation the Anteroposterior radiographs; **(h)** two months post operation the Lateral radiographs.

**Figure 5:**
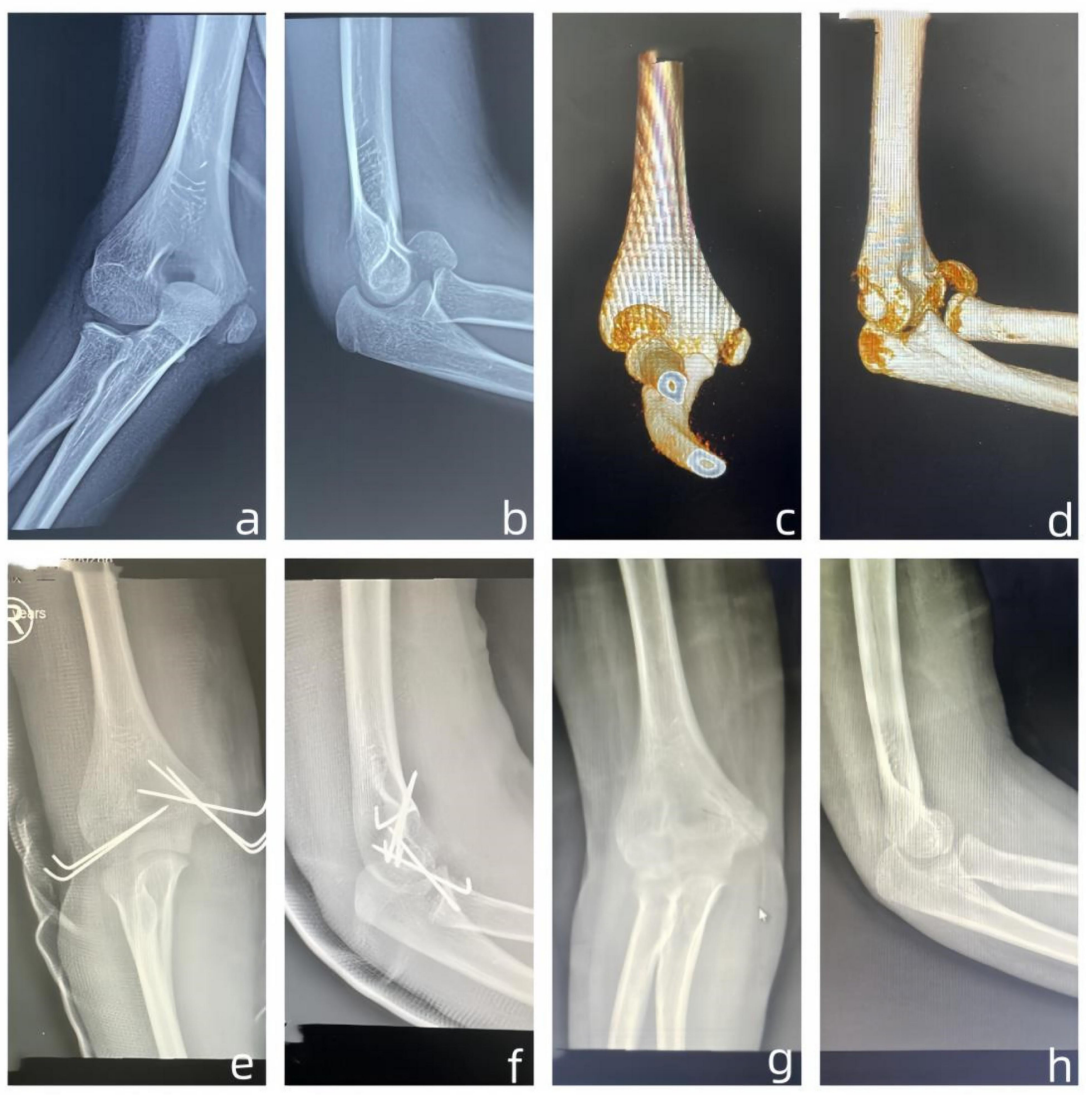
**(a)** One 13 years girl, traffic accidents, 1 day after injury the Anteroposterior radiographs; **(b)** 1 day after injury the Lateral radiographs; **(c)** 1 day after injury the Anteroposterior Three dimensional CT; **(d)** 1 day after injury the Lateral Three dimensional CT; **(e)** 1 day post operation the Anteroposterior radiographs; **(f)** 1 day post operation the Lateral radiographs; **(g)** 4 months post operation the Anteroposterior radiographs; **(h)** 4 months post operation the Lateral radiographs.

The MELAINE-flexion type involved thirteen cases (male7, female6), left side 6 and right side 5. Injury mechanisms consisted of one car accident, one fall from height, six simple falls, and five sports injuries. All cases received surgical open reduction. This group included MEP (type II 3, type III 10), fixed with Kirschner wires 10 and absorbable screws 3. There were three capitellar fractures (type II 1, type III 2), fixed with absorbable screw 1 and Kirschner wires 2. Additionally, LC 10 (type III 4, type IV 2, type V 4) were all fixed with Kirschner wires, following closed reduction 6 and open reduction 4 (Figures 4, 5).

**Figure 6:**
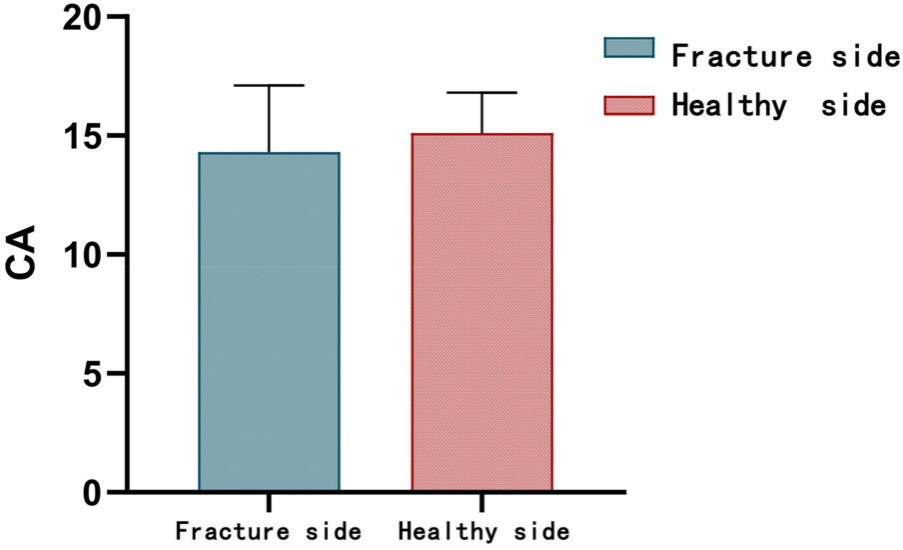
The performance of CA.

Radiographic evaluation confirmed fracture union in all patients at a mean of 5.8 ± 1.15 weeks (range 6–10 weeks). At final follow-up, the fracture side showed mean elbow flexion–extension and forearm pronation–supination arcs of 139.2° ± 9.4°, 4.5° ± 3.4°, 75.8° ± 8.1°, and 79.6° ± 8.2°. The carrying angle on the injured side measured 14.3° ± 2.8°, respectively, compared to 15.1° ± 1.7° on the healthy side (*p* = 1.78) (Figure 6). No cases of joint stiffness or surgical infection were observed. Four patients developed Kirschner wire irritation syndrome, which resolved following wire removal. There were no instances of delayed union, nonunion, malunion, heterotopic ossification, cubitus varus, fracture displacement, re-fracture, implant failure, or limb length discrepancy. Four nerve injuries occurred pre-operation: involved the ulnar nerve 3 and median nerve 1, all were contusions without complete disruption.

## Conclusion

The incidence of MELAINE is rarely documented. A multicenter study reported 11 cases in children aged 6 to 14 years, representing an incidence of 2.55% (11/431) among elbow fractures. In our series, the incidence was 2.21% (21/949). This diagonal injury pattern was first described in the knee as the “reverse Segond fracture” and has since been reported in other hinge joints. The mechanism typically involves axial compression combined with varus or valgus stress, resulting in a concomitant injury on the contralateral side.

**Figure 7:**
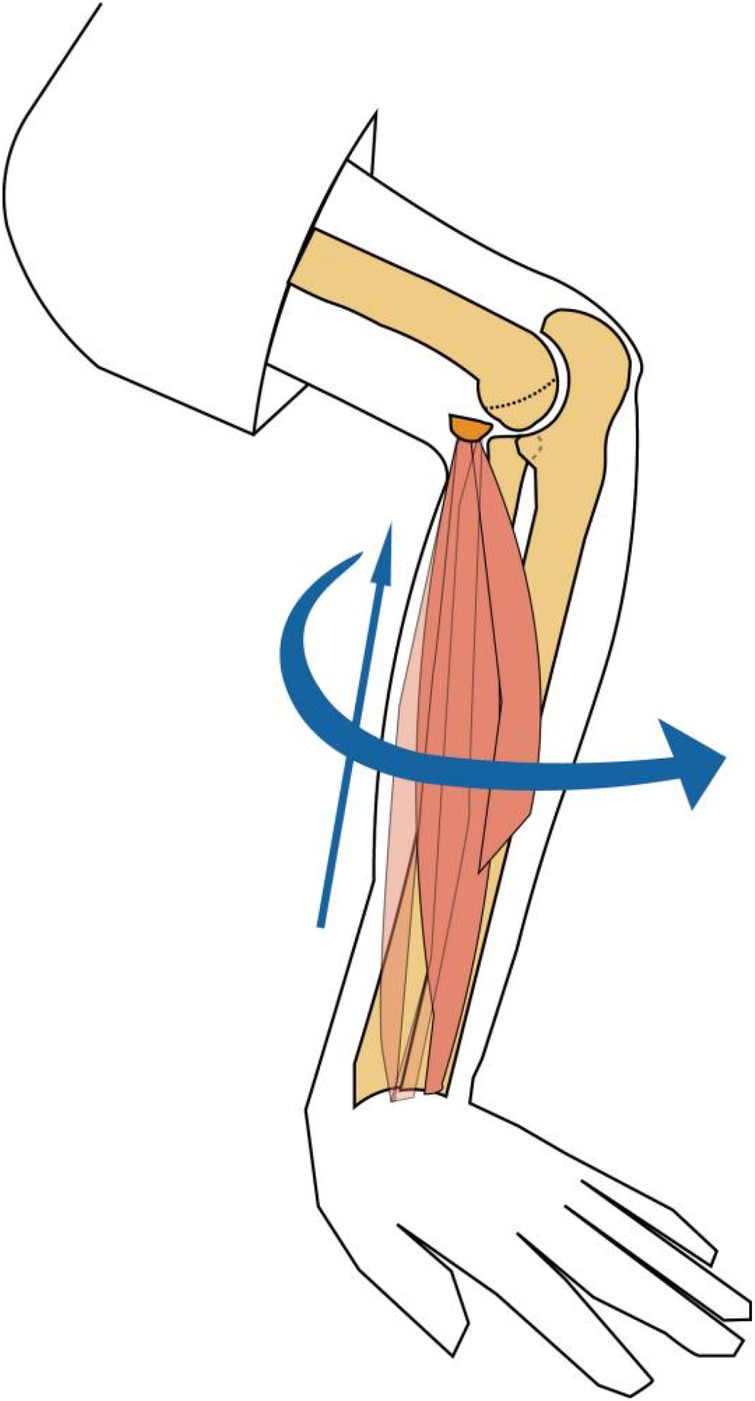
Schematic diagram of the pathogenetic mechanism.

MELAINE is first classified into extension and flexion types. The primary injury mechanism involves valgus stress at the elbow, leading to a medial epicondylar fracture. In the extended position, a longitudinal compressive force transmitted through the forearm fractures the relatively weak radial neck (7). When the elbow is flexed, valgus and axial compression forces act simultaneously on the limb. With the palm supporting the ground, the longitudinal force from the forearm transmits to the capitulum and lateral humeral condyle, potentially resulting in lateral condyle or capitulum fractures (Figure 7). Although the flexion type of MELAINE has been mentioned previously, it has not been systematically summarized. Lu et al. (7) reported a case of a medial epicondylar fracture combined with a capitellar fracture and radial neck fracture, proposing that this combination also follows the MELAINE mechanism. They suggested that characteristic impaction occurs between the capitulum and radial head following valgus stress; sufficient force may cause a simultaneous capitellar and proximal radial fracture, termed a “kissing injury.” We observed two such cases in our series, and a similar fracture pattern was reported by Kun et al. (11). Within MELAINE cases, medial epicondylar fractures were mostly complete (Type II: 4; Type III: 17). Displacement in avulsion fractures was significantly more severe than in compression fractures, a finding consistent with reports by Li et al. (11) and Lu et al. (7), suggesting the medial side of the elbow likely experiences force before the lateral side. This explains why lateral condyle, radial neck, and capitellar fractures in MELAINE, particularly those without significant displacement, are occasionally underdiagnosed. A similar diagnostic challenge occurs with anterior cruciate ligament substance tears or avulsion fractures, which are frequently associated with meniscal injuries and also have a relatively high omission rate (12).

Elbow injuries are common in pediatric orthopedics (13–15). Clinically, when an elbow dislocation is present, physicians typically focus on the multiple associated injuries (5). However, in the absence of dislocation, concomitant fractures in the same limb carry a risk of being missed (11). In pediatric MELAINE injuries, we observed an initial radiographic omission rate of 23.80% (5/21). For instance, when a clearly displaced MEP is identified on routine outpatient radiographs, an accompanying non-displaced RN fracture may be overlooked. In our series, MEP fractures were missed twice on initial x-rays, capitellar fractures once, and RN fractures twice.

**Figure 8:**
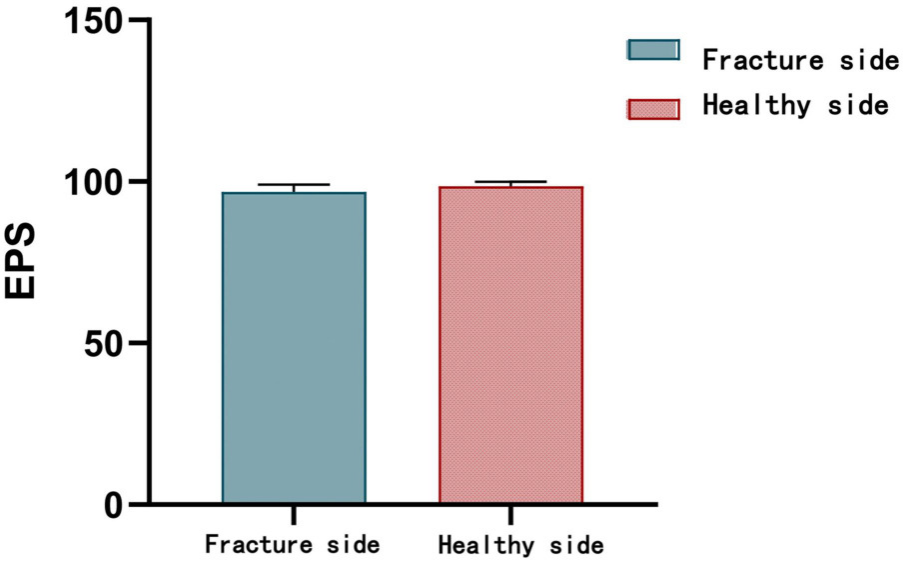
The performance of Elbow Performance Scale score (EPS).

MELAINE involves two or more concurrent fractures of the elbow joint. Most documented cases of such combined fractures are accompanied by elbow dislocation and carry a relatively poor prognosis, with a complication rate of 34.8% (8/23) and postoperative elbow stiffness in 21.7% (5/23) of patients (16). In contrast, diagonal fractures in children without elbow dislocation, whether classified as MELAINE or LAMEINE, generally have a favorable prognosis (6, 7). In our series, most patients achieved an “excellent” (*n* = 18, 85.7%) or “good” (*n* = 3, 14.3%) EPS rating (Figure 8). The fracture-side carrying angle measured 14.3° ± 2.8°, compared to 15.1° ± 1.7° on the healthy side. Lu et al. also reported good outcomes for 12 cases of the MELAINE-extend type, with radiographic union occurring in an average of 6.3 ± 1.2 weeks (range 4–9) and excellent results in 11 patients (91.7%) (7). These findings are broadly consistent with our results. Treatment outcomes show no significant difference compared to isolated MEP, RN, LC, and capitellum fractures (9, 17–22). The incidence of complications is also not significantly increased (Table 1).

**Table 1:** Comparative analysis of “diagonal lesions” of the elbow: lateral to medial diagonal injury of the elbow and medial to lateral injury of the elbow.

Variables	MELAINE			*t*/*χ*	*P* value
Classification	Flexion		Extension		
Fracture	MEP + LC	MEP + capitellum	MEP + RN		
Age, years	9.9 ± 2.1	9.5 ± 0.31	8.4 ± 1.0	−6.31	>0.05
Sex, *n* (%)				0.451	0.463
Male	3 (60%)	2 (66.7%)	7 (53.8%)		
Female	2 (40%)	1 (33.3%)	6 (46.2%)		
Side, *n* (%)				0.363	0.791
Left	4 (66.7%)	2 (66.7%)	7 (53.8%)		
Right	2 (33.3%)	1 (33.3%)	6 (46.2%)		
Injury mechanism (%)					
Traffic accidents	1	0	1		
Falls from height	0	1	2		
Simple falls	5	1	3		
Sports injuries	4	1	1		
Collision	0		1		
Fracture distribution				0.465	0.331
MEP	type II 2, type III 6		type II 3, type III 10		
RN			type II 2, type III 3,		
type IV 3					
LC	type III 4, type IV 2, type V 4				
Capitellar fracture		type II 1, type III 2			
Elbow Performance Scale (EPS) score, *n* (%)				0.336	0.112
Excellent	8	2	8		
Good	2	1	0		
Poor	0	0	0		
Complication					
Nerve contusion	0	ulnar nerve: 1; median nerve: 1	ulnar nerve: 2;		

MEP, medial epicondyle of the humerus; LC, the fractures of the lateral condyle of humerus; RN radial neck fractures.

This study has several limitations. First, as a retrospective analysis, it lacks detailed rehabilitation outcome data for the children. The cohort was identified from surgical records and procedural codes, potentially omitting patients with similar injuries managed non-surgically. Second, the follow-up period was relatively short, and some outcomes may improve with longer observation. The EPS score was employed as the outcome measure because functional outcomes were absent from the medical records and it has precedent in the literature. Reliably assessing fracture healing time also presents a challenge in a retrospective design. Variations in follow-up intervals, and consequently in the available radiographic examinations, inevitably influenced the healing time estimates. Finally, the range of motion were derived from surgeons' notes in outpatient records, offering a subjective comparison to the contralateral elbow rather than an objective measurement.

### Conclusion

Period was relatively short, and some outcomes may improve with longer observation. We employed the EPS score as the outcome measure because functional data were absent from the medical records and this metric has precedent in the literature. Reliably assessing fracture healing time is also challenging in a retrospective design. Variations in follow-up intervals, and consequently in the available radiographic examinations, inevitably influenced these estimates. Finally, the range of motion measurements were derived from surgeons' notes, providing a subjective comparison to the contralateral elbow rather than an objective assessment.

## Data Availability

The original contributions presented in the study are included in the article/Supplementary Material, further inquiries can be directed to the corresponding author/s.
